# How to acquire and use information about pupils to improve the transition from primary to secondary school?

**DOI:** 10.1016/j.heliyon.2023.e16257

**Published:** 2023-05-12

**Authors:** Marlau van Rens, Wim Groot

**Affiliations:** Maastricht University School of Business and Economics Maastricht University UNU-MERIT / Maastricht Graduate School of Governance P.O. Box 616 6200 MD, Maastricht the Netherlands

**Keywords:** Transition-process, Primary-secondary school, Stakeholders, Mentor-support, Freshmen

## Abstract

The transition from primary to secondary school can affect children’s school and work careers. Mentors at secondary school guide the children through the transition process. For this, they need support from the children, their parents, and the primary schoolteachers. We interviewed 17 mentors from secondary schools in the Netherlands to investigate whether they acquire the information they need and how they value that information. The results show that mentors play an autonomous role, are insufficiently aware of the experiences of primary schoolteachers and dissatisfied with the overall educational report by the primary school. Direct contact with primary education teachers is greatly appreciated but often does not happen.

## 1 Introduction

Teachers play an important role in guiding and supporting children in the transition from primary to secondary school. Mentors at secondary school are designated to assist the children of ‘their’ class through this process and to provide the social-emotional and academic support children need to make a successful transition. Every year, after the summer break, many children all over the world make this transition and although school systems differ, the challenges and threats are broadly similar [26]. Most children initially experience anxiety, stress and a decrease in motivation [43,58] which, if left unchanged, may impair their social-emotional development [35,51,52] and undermine their academic achievement [24].

To support their personal and academic development it is important that children feel cared for by the people in their surroundings, including by their schoolteachers [37]. Healthy interpersonal relationships are a pre-requisite to rewarding learning relationships [50]. Individual help and support by teachers are critical for a meaningful contribution to students’ adaptation to a new school [[17], [45]].

When children make the transition to secondary school new relationships need to be built with their new teachers [50]. However, once at secondary school, children perceive that they receive less support from teachers [50]. Teachers at primary school spent more time with their class than teachers at secondary school and therefore have more opportunities than secondary schoolteachers to foster caring relationships on which to build academic confidence [10]. In contrast to the need of children for support, their relationships with secondary schoolteachers are less personal and more controlling [10]. Therefore, some children -especially girls - may experience difficulty in developing new social relationships. Boys, more often than girls, appear a decline in positive school experiences [50,53]. Moreover, contrary to their expectations before the transition, children experience fewer demands on their cognitive skills at secondary school [25]. That seems to contradict expectations of secondary schoolteachers that children will have learned to take more academic responsibility for their work [10].

The class-mentor has an important role in the guidance of ‘his/her’ class and can contribute to a smooth transition by assisting and supporting the children through the transition process. However, the mentors cannot take on such a task in a vacuum. They need support as well, sufficient to properly perform the role of mentor. They also need to be able to acquire the information necessary to determine how to adequately deal with the demands and desires of ‘their’ children [25].

Academic research about the transition, on the one hand, confirmed that teachers play an important role in guiding and supporting children but on the other hand has paid little attention to the teachers’ perspective about what is required to accomplish that important role. In this study we investigated how mentors at secondary school acquired information about their pupils and whether they were provided with sufficient resources and time to use this information to improve the transition. We were interested if (and which) information about the children making the transition reached the mentor and, in particular, if this information included statements from the children themselves. We wondered if -and how-mentors use the information they obtain, especially that provided by their pupils.

This study presents the views about the transition of 17 mentors of freshmen in secondary education in the Netherlands. Information was collected via focus-group discussions and is used to investigate whether mentors acquire information about the needs and expectations of their future pupils with respect to the transition and how they use this information. First we describe how our study relates to previous research and explain the context of the study. Next we describe the methods that have been used and data that have been collected. Finally, the results of our study are presented and discussed.

This study provides new insights as – to the best of our knowledge - it is one of the first to present the perspective of the mentors at secondary education about the transition process from primary to secondary education. The results of this study are applicable to a broader context than just schools in the Netherlands because although there are differences in education systems [29,30], in almost all countries in Europe and elsewhere children transition from primary to secondary education and many countries have some sort of educational tracking. At a certain age, pupils are assigned to different levels of secondary education based on their abilities and interests. There are differences however between countries with regard to the age at which the transition from primary education to secondary education and the educational tracking happen. However, this does not limit the relevance of our study as all systems have in common that at some point children have to get used to a new school environment and this transition can result in adjustment problems [33,36] and mentors involved want to be well informed about the new students they are about to receive to ensure a smooth transition.

## 2 Literature review about support as perceived and experienced

As mentioned before, education systems differ in the age at which the transition from primary to secondary school takes place. European education systems can be divided into three types: 1. One common education system, where education is the same for all students from the beginning to the end of compulsory education. There is no transition between primary and secondary education. All pupils attend the same general secondary "R29_Jallade" education. 2. Common core curriculum provision. After primary education, all pupils move on to lower secondary education where they all follow the same curriculum. 3. Differentiated lower secondary education. After primary education, pupils follow various educational pathways or different levels of education, either at the beginning or after the lower years of secondary education [11]. Regardless of the age at which students make the transition and the educational system of which they are part, after the transition children must adapt to a new school and a new social environment [33,36,55]. In addition to the influence of the education system, intercultural differences, for example caused by migration, ethnicity and national policy, also can influence education and therefore also the transition [[14], [29], [44]].

Transitions can affect children's educational and well-being outcomes because children may lose their school friends and feel insecure about the relationship with their future teachers. To ensure that the children adjust to their new secondary school environment and successfully meet the challenges that occur, support is important [1]. The greater the discontinuity the children experience and the less the preparation for secondary school, the more support is needed [1].

Supportive teacher-student relationships can enhance children’s motivation and engagement, promote social behavior and improve academic attainment. These supportive relations are associated with lower levels of anger and depressive symptoms, as well as with enhanced adjustment and self-esteem. Less supportive teacher-student relationships can cause children to lose interest in learning [57].

To contribute to a successful transition, by helping children adequately, teachers need to be supported themselves as well. Class-mentors, in particular, have to be given sufficient background information about the needs of the children of their class [25]. Teachers also need opportunities to work together and learn from each other as part of their professional development [3,39].

Help can be provided by the feeding primary schools, the children and their parents, by sharing all the information they find important for a successful transition with the mentor at secondary school [[22], [41]].

These stakeholders may have perspectives and interests which may differ from the perspective of the mentor. Below we describe, based on the literature, how each stakeholder, can provide support for the educators at secondary school and the way that teachers, especially mentors, have been shown by previous research to handle the stakeholders’ support.

### 2.1 Providing support during the transition process

• Primary school

An important stakeholder and source of information is the feeding primary school. Because pre-transition factors may influence children’s post-transition difficulties, primary schools provide the secondary school with background information about the children. Providing information can reduce children’s fears about their future secondary school. Their anxieties include, for example, being the youngest, bullying, discipline, new subjects and homework [42]. However, the information passed on by primary schools usually addresses other matters such as children’s academic performance and test results, their personal strengths and weaknesses and their behavior.

Secondary schools generally accept children from multiple primary schools, not all of which provide information in the same way: it can differ in both content and detail. Sensitive information about children’s background is usually provided verbally and needs to remain confidential (on a need to know basis) [42]. Teachers, children and parents have different perspectives about what they believe are important issues in the transition [54]. Parents and children are concerned with personal and social issues in the here and now, while teachers seem to be more preoccupied with institutional initiatives such as delivering the curriculum. Teachers also take a longer time perspective scrutinizing children’s development over a longer trajectory [50]. Surprisingly teachers very rarely refer to personal skills and do not seem to value the development of children’s own coping skills, for example as they learn to cope with stress, or take control over their lives and become less dependent on organizations and extern structures [50,59].

• The children

The second, -perhaps most important-source of information, are the children themselves. Children who make the transition, bring experiences and understandings from their former school to their new school. These perceptions do not necessarily correspond to the new school environment. During the transition the role of the children may change but children do not necessarily appreciate this [50].

Although children need support, they are also capable of informing their mentor about their needs and expectations. For educators, including mentors, children’s input can make a valuable contribution to understanding how children experience their school live, and what is important to them [34]. In contrast to primary school, children at secondary school develop fewer close relationships with their peers and teachers and may tend to conform more to group norms [49]. At their new secondary school children have to deal with multiple teachers. It may be difficult to build personal relationships with all of them [4].

Knowing that social relationships with teachers and peers can contribute to a sense of community in the school environment leads one to expect that teachers will hear children’s voices. Unfortunately, this is not always the case. Not all teachers find it important to support children during the transition process or to talk with children about their needs or expectations. Hopwood et al. [25] found in a recent Australian study that 42% of the secondary schoolteachers (and 60% of the primary schoolteachers) did not prepare ‘their’ children. Only 28% of secondary schoolteachers talked with children about what to expect.

For many children, one of the biggest demands in the transition is making new friends and building new peer-relationships [51]. Feelings of vulnerability can be reduced if the children feel they are accepted as part of the group [6]. Evangelou et al. [12] found that support in form of the perceived friendliness of children and having children from their primary schools in their new classes, led to positive transition experiences. According to Evangelou et al. [12], this aspect unfortunately, receives relatively little attention from schools.

• The parents

Generally, the perceptions of parents and children do not differ much. As described previously, parents are mostly concerned about the social emotional challenges their children face in the here and now [51]. However, the aspirations of the parents are not always matching to the aspirations and/or the cognitive capacities of the child [32]. A successful transition necessitates the involvement of parents: as parents and as role models. They are uniquely positioned to motivate their children on higher achievement. Communication between parents and teachers should enable them to work together to prevent transition problems or to intervene appropriately when problems do arise [1]. Therefore, schools should use the opportunity to engage parents in a partnership in the transition process, to help their children to develop positive bonds to home and school, and a to construct a view of themselves in the social context [9,38]. Unfortunately, parental involvement is not always obvious. Research shows that 1 in 10 parents in the Netherlands never visit their child's school. In the United States almost a third of the parents never visit the school and other countries are in between [38]. The variation in parental involvement seems to be related to SES and ethnicity of the parents on the one hand and the school's attitude towards parental involvement on the other. Immigrant parents usually have a marginal position and involvement within the school [38]. Social mixing can contribute to the enhancement of wellbeing and social mobility of vulnerable population groups like children with parents with low SES but also, for example, children from Roma populations. However, desegregation alone is insufficient, and further policies are required to increase socio-spatial integration and deal with extreme poverty [2,5].

## 3 Methods

### 3.1 Research context

This study investigates if and how secondary school class mentors obtain and use information to support children through the transition process and the extent to which they are facilitated to do so. The research was not submitted to the ethics committee as this is not mandatory for this kind of research. Maastricht University has committed itself and is bound to the Dutch Code of Conduct for Research for Scientific Integrity (https://doi.org/10.17026/dans-2cj-nvwu). We adhered to this code of conduct in conducting and reporting our research.

Seventeen class mentors in grade seven, from four larger secondary schools located in the southeast of the Netherlands, participated in the study and were interviewed. Sixteen mentors were interviewed during four focus-group discussions; one mentor was interviewed separately. Although the study was based on the experiences of these regional Dutch secondary schoolteachers it provides insights that can be useful for other educational settings in the Netherlands and elsewhere. After all, despite the differences in school systems, children experience remarkably similar challenges and threats during the transition.

Based on their capabilities and interests and on the advice and the test results from the primary school, a choice is made by the children and their parents for one of the tracks of secondary education. In the Netherlands secondary education is divided into four tracks: practical education (PrO), preparation for intermediate vocational education (VMBO), upper-secondary education (HAVO) and preparation for academic education (VWO). Larger school communities often provide multiple tracks of secondary education.

This study is part of a larger project which followed 371 children in the southeast of the Netherlands, from the final term of primary school to the end of the first term of secondary school [[52], [53], [54]] (Authors 2018; 2019; 2020) [55], (Author 2020). Consequently, the geographical location of the secondary schools for the current study was the same as the sampling of the children’s part of the project. Other selection criteria were that the schools had to be representative of the range of secondary education in the region and had to offer different tracks of secondary education and belong to different school boards.

The participating schools are school communities, containing the three secondary school types VMBO, HAVO and VWO. The number of pupils of the schools varied between 1044 and 1954. Except for one school, all schools have a Roman Catholic signature. Three schools belong to three different large school boards; one school has its own school board (www.scholenopdekaart.nl). One of the schools (school 2) offers specialized education for children with special educational needs (SEN). This provides a bridge between primary education for children with SEN and secondary education. In order to be accepted into this school the children must have a referral which is issued by a regional alliance. To be considered for this referral the children have to go through several observation periods and tests. If the intake level is too high for a child, school 4 has made agreements with a school for practical education (PrO) to let their students enroll there.

### 3.2 Participants

After selecting the schools as described above, the grade seven department manager in each secondary school was contacted. All department managers recruited at least four mentors from different school types to participate in the focus-group discussion. All mentors were teachers. The mentors were asked to participate in a focus-group interview that would last at most 75 min. They differed in experience as teachers and as mentors. Education is a predominantly feminine occupation in the Netherlands, so the focus groups had a higher proportion of women than men participating. To ensure confidentiality, each teacher was allocated a letter which replaced their name. Table 1 shows the characteristics of the mentors from the four schools, who participated in the focus groups.

**Table 1:** Characteristics of interviewed mentors.

M = Male F= Female	School	Teacher	Years teacher	Years mentor	Subject	Mentor class	Class size
M	1	A	41	40	German language Math	MAVO/HAVO	29
F	1	B	27	27	English Language	HAVO/VWO	22
F	1	C	3	1	Math	MAVO/HAVO	28
F	1	D	30	3	Dutch language	Gymnasium	26
F	1	E	19	12	Geography	MAVO/HAVO	27
F	2	F	5	4	English Language	VMBO	18
M	2	G	5	4	Economy	VMBO	26
F	2	H	13	11	Dutch Language	VMBO (SEN)	18
M	2	I	8	6	History	HAVO/VWO	26
F	3	J	29	27	Dutch language	VWO	31
F	3	K	28	28	Music	VMBO-T/HAVO	32
M	3	L	26	26	Gymnastics	VMBO	31
F	3	M	5	4	English language	VMBO	31
F	4	N	13	13	Dutch language	HAVO	22
F	4	O	17	17	Math	VMBO	15
F	4	P	14	14	Biology Gymnastics	VMBO	17
F	4	Q	15	15	Dutch language	VMBO	22

Note: the tracks at secondary education are: practical education (PrO), preparation for intermediate vocational education (VMBO), upper-secondary education (HAVO) and preparation for academic education (VWO; gymnasium).

### 3.3 Data collection

We based the qualitative design of this study on Grounded Theory, as first described by Glaser and Strauss [19] in their book entitled: *The Discovery of Grounded Theory: Strategies for Qualitative Research*. Grounded Theory generates theories by creating different concepts from the data collected. These theories are generated from the context to which they will later be applied [19]. In this study the data are generated in the school context.

In order to gain insight into their perspectives, the class-mentors were interviewed at their secondary schools during the spring semester of 2019. All interviews were conducted by the same researcher who acted as moderator. As described above, our research question was: (How) do mentors use information from -or about-students to promote a smooth transition between primary education? To answer this question the moderator asked the following sub questions:

• Mentors inform themselves about new students. Via which people or channels do the mentors get information (are the new students involved in this?)
• What information do the mentors think they need?
• Do the mentors use the information they receive, if so how do they handle this, if not what is the reason for this?
• Do the mentors think they can contribute to a smooth transition?

The focus groups with mentors were conducted until saturation occurred and the focus group interviews provided no new information anymore. Data were collected over the course of four consecutive weeks via four focus group interviews and one individual interview. The mentors were interviewed as a group except for one person who, due to scheduling conflicts was unable to participate in a focus-group. Both the focus group participants and the mentor who was interviewed individually were asked the same questions. The size of the focus-groups varied between three to five people and the interviews lasted from 45 to 90 min. With the consent of all participants, the interviews are audio recorded for later transcription and analysis.

Pursuant to Grounded Theory we transcribed the interviews and applied a coding strategy to the transcribed data. As described below, we analyzed and categorized the data, according to a constant comparative method and summarized them into themes via open, axial, and selective coding [47].

The audio recordings were completely transcribed, using verbatim transcription. The transcripts, were used to describe, summarize and interpret the opinions of the mentors. Similar and different findings were compared across interviews by generated codes, the so called open coding, which includes repeated readings of the interviews, an in-depth, line-by-line analysis of the data and coding data under various categories. Ultimately, we analyzed and combined the codes to develop themes. In Grounded Theory, this is called axial coding. In the axial coding, the categories are linked together. As soon as the interviews no longer provided any new information because saturation had occurred, we stopped conducting any further new interviews. In the final, selective coding, the linked categories resulted in a core category [47]. To ensure the themes were an accurate and valid representation of the interviews during the coding process we reread and reviewed the transcription several times.

**Fig. 1:**
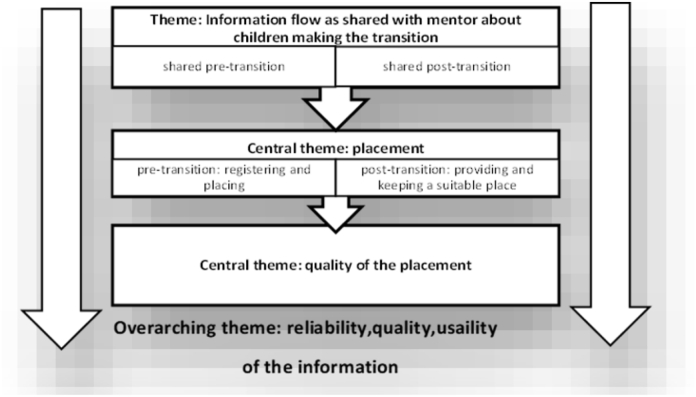
Schematic presentation the process of the coding process strategy.

The theme of the interviews was the information flow, as shared with the mentor at secondary school, about the children making the transition from primary to secondary school. This included information about the academic as well as the social-emotional, development of the children which was capable of being provided by the stakeholders in the transition process: primary school, secondary school, the children and their parents. The interviews yielded a large amount of initial data, which was coded in stages (open coding). By comparing the interviews, using axial coding the information was merged and grouped into two types of information: information provided before the official start of the new school year at 1 August (pre-transition) and information provided after 1 August (post-transition). Both, pre- and post-transition information have a central theme. The purpose of the pre-transition information is registering and placing a child at secondary school, the central theme is the placement of the children. The purpose of the post-transition information is providing (and keeping) children find a suitable place at secondary school where they can succeed in a safe educational environment. The central theme (selective coding) is the quality of the placement. The overarching theme is the reliability, quality and usability of the information (see Fig. 1).

The themes are described from the perspective of the mentor. Being aware of the information leads to more autonomous behavior of the mentor, incidentally before- but usually after the transition.

### 3.4 Advantages and limitations of a qualitative research design

By examining the opinions, behavior and attitudes of the respondents, qualitative methods, as used in this study, can generate knowledge that cannot be obtained by quantitative methods. According to Kelle [31], this method can provide mutual validation of data and findings, as well as produce a more coherent and complete picture of the context of our study. Using focus group discussions can be viewed as contributing to the validity of a qualitative study [8].

#### 3.4.1 Participants and focus group interviews

To answer the research questions of whether, and how, mentors acquire and use information about children to improve the transition to secondary school, we interviewed 16 mentors from four secondary schools in four focus groups and 1 mentor separate. This procedure was intended to guarantee first-hand involvement in, and understanding of, the patterns, structures and rules in the investigated context of the transition from primary to secondary school.

A focus group interview is a technique designed to collect information from a small group of ‘insiders’. The process requires that they answer approximately five significant questions. The advantage of the qualitative method is that it answers important questions and generates many ideas, possibilities and initiatives in a relatively short period of time. Consensus and discussion with regard to the theme, as well as prevailing norms and values, readily become clear. However, this method also has limitations. Focus interviews usually contain a limited number of questions. The method is time consuming - and therefore costly - because several interviews are needed to collect sufficient information. Because the results of the interviews consist of subjective experiences, this may have consequences for transferability.

Managing a focus group interview can be difficult. There is a risk that the participants could influence each other or that one of the members of the group might dominate the discussion [13,16]. To overcome these potential handicaps, the focus group needs a trained and experienced interviewer. Inexperience, subjectivity and inaccuracy in analyzing the data can influence the reliability of the research. Research bias may also arise when the interviewer takes a leading role in the discussion, advises the focus group or makes a judgment about the information from the group.

The investigator who conducted the interviews in our study was trained to lead focus group discussions and formulate the research questions. Furthermore, she was an experienced teacher and mentor in grade 7 herself, so that she was already familiar with the educational context at secondary school.

#### 3.4.2 Objectivity, reliability and validity in qualitative research

To achieve the necessary objectivity, reliability and validity [48], we checked whether the research theme - *‘the information transfer during the transition from primary to secondary education’* - played a role among the mentors involved and the extent to which their views were consistent or different from each other. We asked the mentors to indicate whether they agreed with the content of the transcript and, if that was not the case, to make appropriate adjustments. We also checked whether there was a relationship between the results of the interviews and the results found in the scientific literature.

According to Guba & Lincoln [23], in qualitative research validity becomes credibility and transferability, reliability becomes a thorough contextual description, and objectivity becomes inter-subjectivity. To increase the credibility of our study, the data were documented by recording the focus group interviews and transcribing all audio material. To ensure transparency, we clearly described our research method, the research context, the participants and the data collection.

Because we used a qualitative method to find out about individual, subjective perceptions of the mentors, objectivity is a difficult concept. At best, we can strive for inter-subjectivity which means that the respondents agree with the results of the survey and there is a kind of common truth. In addition, when analyzing the data, the researcher must take her own history and experience into account so as not to skew the results. We asked the respondents in our study to read the transcripts and indicate whether they thought the transcription was correct and whether they agreed with our interpretation of what had been said. Thereafter, the data were analyzed by looking for and finding patterns in the responses of the mentors (i.e., the respondents). For this, we used the Grounded Theory method (GT), which aims at generating concepts that explain the way that people resolve their central concerns. The disadvantage is that the results of our study do not contain statistical data but show the relationship between hypotheses developed on the basis of empirical data [21]. Consequently, validity in GT -and therefore also in our research-, is assessed for appropriateness, relevance, workability and adaptability [[19], [20], [21]].

## 4 Results

### 4.1 Information provided by the stakeholders before the transition to inform the mentor

According to the interviewed mentors, the phase prior to the transition proceeds in a similar way at all schools. The primary school advises children and their parents which education track is the most suitable. This advice is binding for eligibility unless the outcome of the final test at primary school turns out to be higher than anticipated in the school-advice. In that case, the advice can be adjusted upwards. A well-founded school advice therefore not only provides information about the academic development of a child, but also gives insight into their social and emotional development. This is important, because children will confront not only a new learning environment in the secondary school, but also a new social environment [46]. Based on the primary school-advice, parents register their child at the secondary school of their (and their child’s) choice. At all secondary schools participating in our interviews, children were able to indicate with whom they wanted to be in their new class. Children generally like a class with children they know or with whom they are friendly. When they are reluctant to attend the same class as a familiar child, it often has to do with a history of bullying. As one of the mentors during the interviews indicated:

“And then they come to one or two names of children, with whom they want to be in class and they can also indicate if they absolutely do not want to be in a class with someone. For example, with a history of bullying.”

To the greatest extent possible, the wishes of the children are taken into account so that friendships do not have to be broken and bullying can be prevented. According to another mentor, and confirmed by her colleagues, a mentor has to be aware of:

“Bullying that happened at primary school. That's nice if you know that, then you can keep an eye on that.”

As soon as the children are placed at a secondary school, the feeding and receiving schools start handling the administrative work, such as forwarding and receiving tracking documents, preparing the digital tracking system and forming the classes. Most information is exchanged digitally.

At one secondary school, the teachers (as ambassadors) establish direct contact with the teachers of all feeding primary schools to collect information about all placed children.

O explained: “Most of us are ambassadors for a primary school. You are assigned to one, two, or sometimes more primary schools. There you have a conversation with the teacher of grade six, and you get information about the typical children. This information complements the information of the educational-report, because an educational-report says so little. In the individual conversation with a primary school grade six teacher, you get a little more insight about the children.”

From May 25, 2018 only one privacy law applies across the EU. Stronger rules on data protection mean people have more control over their personal data. Retrieving the information orally, allowed the mentors at this secondary school to bypass the privacy law, that prohibits written information from being transferred before 1 August (and only with the parents’ consent). As a result, this secondary school is the only one in the interviews, that already had a complete file on every child before the summer break.

At the other secondary schools, a so-called warm transfer takes place. A warm transfer is a conversation, usually between the teacher of grade six at primary school and the mentor of secondary school, about a particular child. According to the mentors, the secondary school usually takes the initiative on this, although sometimes it is at the request of primary school. Forms of warm transfer, mentioned by the mentors include: telephone or e-mail contact, “speed dating” (short conversations about the future students from several feeding schools), direct contact discussing pupils who need extra attention, direct contact discussing all children. The secondary school mentors regretted that not all primary schools respond to the invitation for a warm transfer to the secondary school.

Apart from the warm transfers and the work by the mentor ambassadors, most mentors do little to actively collect information about their future class until the summer break. They usually receive a brief summary, made by an intermediary such as a team leader or a coordinator, with only the most urgent information about the children of their class. As expressed by J:

“Before the summer break you will only know very important things about your class, such as the information I received about a boy whose mother had died just before the introductory afternoon”.

Following this information mentors may sometimes find it necessary to directly contact the parents or the former primary schoolteacher of the child. In that case they would make a personal appointment.

Mentors do not generally meet or talk with individual children prior to the new school year, except sometimes when it concerns children with SEN. In the Netherlands, all children are supposed to attend a school that provides an education suited to their talents and capabilities. It is required by law that all schools offer an appropriate place to children who need extra assistance. About one in every five pupils needs extra assistance at primary school (https://www.government.nl/topics/primary-education/appropriate-education-at-primary school). With this in mind, schools should expect to adapt their teaching to the individual child's development. At one of the four secondary schools participating in our study, the mentor had an individual meeting with the children who had extra assistance at primary school. One of the mentors indicated that it would be her preference, if she was given the time, to have a conversation with all the typical students before the transition.

Mentors meet their class for the first time as a group in June, before the summer break, on an introductory day or afternoon, generally designed and organized by the future mentors of the children of grade 7. Children with SEN who enter the special educational facility have a referral which is issued by a regional alliance, as described above. These children will have had several tests. As a result, the file of SEN children is complete before the transition. Those children also follow an introduction program which is more structured than the regular program.

### 4.2 Reliability, quality and usability of the pre-transition information

The quality of the school advice does not guarantee a proper transition for two reasons. Firstly, the school advice does not always take into account all the skills, cognitive and non-cognitive, that children need to make a successful transition. Therefore, the quality of the transition may depend on factors that are not necessarily all captured in the school advice, such as the extent to which an individual child is able to adapt to a new environment. Switching from a small, self-contained primary school to a large, more heterogeneous secondary school requires different social and academic skills [24,40]. Secondly, there are personal qualities that may affect a child’s ability to adapt to a new environment, regardless of the school, track (academic or vocational) or level of education, such as independence, motivation and perseverance. Compared to primary school, in secondary school the children are given more freedom and face greater challenges. They also are expected to be more self-sufficient.

The information, mentors receive about their class prior to the transition, includes the school-advise, sometimes a warm transfer – i.e. a meeting - with the grade six teacher. They also receive transfer file (which is usually incomplete) containing the educational-report and the results of the final central test that almost all children take at the end of grade six. There is no uniform school-advice. Schools do not give the advice in the same way and parents play a role in the development of this discrepancy. G explained:

“The general picture in society is that the VMBO is still the ‘dregs’ I do not want to say it, but many parents see it that way. I think, because this is the way they speak in primary school: “Yes my daughter or son goes to HAVO. Then that is seen as better than a VMBO student”.

His colleague added:

“This year we had quite a few students, from theoretical HAVO, who actually did not have the regular HAVO level and who were just fine VMBO-T students. But their parents really had a serious influence on the placement advice. So actually the primary schools were more or less, -forced is perhaps a little harsh word for it, but let’s say exhorted- into giving them an advice for a level higher. So these children are actually working above their level and experiencing a very false start in secondary education”.

At another secondary school the mentors also have reservations about the reliability of the school-advice. They confessed that nowadays the lowest VMBO level is hardly ever advised. These children no longer seem to exist, according to the mentors, because there used to be four classes on the lowest level at their school and now there is only one class. The mentors admitted:

“We see that schools cannot give a completely unprejudiced school-advice, because they are partly judged by it”.

According to the mentors the school-advice level of the children who need extra support is often too high, because teachers at primary school do not take into account the environment in secondary education which places higher demands for children’s independence. One mentor disclosed that in his regular class of nineteen children, twelve children required special care by a special teacher. The mentors were especially regretful that the attention these children demand is often at the expense of that which could otherwise be given to typical children.

Surprisingly, despite the careful placement policy of the regional alliance, even the advice of children with SEN is not always reliable. In particular, the advice by the teacher of the special primary education school is often incorrect. The teachers at schools for special education do not take into account that regular education is not able to support the students in the same way as happens in special education because they do not have the same resources. H said:

“The teachers in special education very quickly think that students have a high level. They look at the children through a different lens then the one they should be using with children getting a VMBO-T / HAVO advice”.

The mentors were all satisfied with the added value of the warm transfer and the quality of the data transferred by the primary schoolteachers during that consultation. They appreciated the direct contact with the grade six teacher at primary school as the most valuable contact and regretted that some primary schools have abolished this or do not respond to the invitation of secondary school for a warm transfer.

The quality of the transfer file varies per school and is usually incomplete. The completed file is available after the start of the new school year at 1 August.

### 4.3 Information from the stakeholders after the transition to inform/support the mentor

Some children are vulnerable to a more difficult transition due to their background (e.g., lower socio-economic status, socio-ethnic origin) or individual characteristics (e.g., gender, behavioral or learning difficulties) [1,56,58]. Adjustment problems (especially those experienced by vulnerable children) may stem from poor preparation for the transition, sometimes combined with little or no support during and/or after the transition [35,55]. On the one hand, mentors believe it important to give children a fresh start at secondary school, so they do not believe it is always necessary to hear all the details of a child's past. On the other hand, the mentors aim to prevent false starts, so they actually want to know the things that are really important, to prevent adjustment problems at secondary school. N said:

“I think it is nice to meet the student open-minded. Therefore, I don't want to know all the ins and outs. I think it's nice indeed to have the possibility of inquiring and making contact about situations if you get stuck in it”.

F, a mentor at another secondary school, expressed something similar:

“I prefer not to be influenced so much in advance. I would rather be open and unbiased when I meet with a student for the first time, but if there are really urgent matters that you really need to know beforehand, then I would like to know”.

The mentors were all in agreement that there was certain pre-information, they would like to know, however, such as notification about a difficult home situation, about certain disorders or about a past history of bullying or suffering from bullying.

After the transition, it appears that some stakeholders are unwilling to provide information to the receiving school. Unfortunately, it is not obvious that the ‘really urgent matters’ are always known by the secondary school. As a result of the privacy law, documents are only provided, if the parents give permission, and only after 1 August, the official start of the new school year, and not before. Until then essential information is mostly withheld. For example, notification about a disorder or a special approach, is sometimes not shared with the secondary school because parents and/or primary schools want to prevent a child being rejected at secondary school or because they believe the child deserves a new chance and a fresh start. J tells:

“I have had the experienced where a parent had deliberately chosen not to pass on the diagnostic report of a child with autism to secondary school, because she wanted her child to get a fresh start. Well, then you first run into many problems and finally the truth comes out. It appears that there are indeed suggestions about how to deal with this child”.

G: “What we have noticed in recent years is that we receive students who all get a chance to prove themselves at a higher level than the school-advice indicates”.

Mentors are facilitated by being allowed extra in-class-hours (1–3 h per week) and non-class-specific-hours (60–80 h per year) to perform their mentor tasks. The in-class-hours are meant to perform tasks in- or related to the classroom, the non-class-specific-hours are for tasks outside of class. One secondary school clustered their available meeting hours and used them for a weekly team consultation. During this meeting, grade seven mentors concentrated on making an inventory of the problems they encountered, and on finding, implanting and evaluating a suitable solution for these problems together. All mentors complained that they did not have enough time to perform their mentor tasks. They accepted the time-pressure because they unanimously agreed that they found mentoring to be personal rewarding. F:

“I really like being a mentor, I would rather be it than not”.

The most common ways that mentors receive information about the class or to gain knowledge about the students, are: orientation days, supporting students with SEN, a transition program, parent-child information sessions, parent-child talks or receiving information or feedback from the primary school. Secondary school mentors have an active autonomous role in designing and organizing the transition program and in contacting and giving feedback to the other stakeholders (parents and primary teachers) with regard to student information. To ensure a successful transition, the mentors all said they most appreciated the direct contact with the primary school grade six teachers, because that was the most valuable contact. Despite this, the interviewed mentors doubted whether grade six teachers at primary school were sufficiently aware of the educational practices of secondary education. L:

“I think their view about us, how we do things here or do things differently, I think that the view is limited”.

D: “I worked in primary education myself for 26 years - until recently – and as a primary schoolteacher you cannot imagine teaching and mentoring in secondary education. It is a big difference”.

Mentors also wondered whether primary schoolteachers were aware of how much they could contribute to preparing children for the transition process by helping them to develop the level of independence they needed to handle the demands of secondary education. In the experience of mentors at secondary school, there was quite a difference in the way children were prepared and supported before the transition. L wondered about the lack of basic skills:

“If they all have to listen to something at the same time, it seems as if they can no longer do it or no longer learn it. Children sometimes look at you as if they are wondering: but why do we have to listen to you with 25 people at the same time?”

According to the mentors, the role of the parents is not limited to support during the transition process. Parents should also play a role in preparing children for a smooth transition. I said he does not think that learning skills, which are necessary to prepare children for secondary school, should only be linked to primary school; such skills are a matter for the entire process of education. G and H agreed.

I: “It just depends on what the parents have taught their children, whether they are able to take responsibility for their own actions or not. I cannot necessarily link that to primary school”.

G: “What I notice is that the students are becoming more and more pampered and more outspoken. I notice that with parents but also with students, and there are indeed more students that you really have to steer towards social skills”.

I: “And you also see here in the breaks whole hordes of pupils, sitting together at the table, who do not exchange a word” …

H: “No, not a word”.

### 4.4 Feedback to the stakeholders: awareness, and experiencing safety and success

Experiences in the first year of secondary education can substantially influence a pupil’s decision about whether or not to drop out [7]. Primary schools and the secondary schools are interested in a smooth transition, because this confirms their ability to properly anticipate the student’s capacity to make the necessary changes (in the case of the primary school) and to adapt to the new circumstances (in the case of the secondary school) [32]. The mentors acknowledged the value and importance of improving the transition brought about by awareness of the practices used by the teachers of their feeding schools. All secondary schools provide some feedback about their experiences to the supplying school. The way they give feedback varies. Some mentors have personal contact with the primary schoolteacher, others give feedback via the person who represents them at the feeding primary school. One school initially invited all primary schoolteachers of grade six of their feeding schools for feedback. Unfortunately, to the regret of the mentors, this process has been stopped because of a lack of time of the primary schools.

The way in which secondary schools provided feedback to the parents is comparable. Feedback was provided by parent–child-mentor meetings that were prepared by the mentor with the children. The duration of the conversations varied per school from 10 to 30 min. The purpose of the conversations was to clarify whether the child was enjoying school and developing, both at home and at school, as may be expected. The mentor plays an active role in the contacts with parents. According to the mentors, parents seldom take the initiative to share their experiences, except when they have negative comments. Parents generally leave it to the mentor to contact them.

Independent of each other, all mentors agreed about the purpose of their efforts during the transition process. In collaboration with all stakeholders, they focused primarily on creating a safe educational environment with sufficient personal attention so that all children could gain success in the experience. G expressed it as follows:

“I believe that a student who starts secondary education must gain success”.

H agreed: “Yes, absolutely”.

G: “And what you very often see is, for example, the students who start in the VMBO-T/ HAVO classes- we have this year, about ten children who have been put on a lower track- all of whom have been receiving unsatisfactory marks, and now all of them are suddenly performing satisfactory, suddenly showing what they can do”.

H: “Yes”.

G: “And who are much more enthusiastic in life and are happier more positive now”.

## 5 Discussion and conclusion

Mentors play an important role in guiding and supporting the children in their class through the transition from primary to secondary school and are in a central position to provide the social and academic support the children need to make a successful transition [25]. To enable mentors to support the children they need support -at the very least, in the form of information about the children in the class-because appropriate teacher support leads to greater student success [18]. We examined, from the mentor's perspective through focus interviews, how mentors are fed information both before and after their class makes the transition from primary to secondary school and how they are facilitated in time and resources. The mentor's actions, in response to the information, was also investigated.

The mentors we interviewed were provided with information by the parents and/or children and the primary school of the children. With one exception, children with SEN, there is virtually no communication with the other stakeholders in the transition process. Active child-participation is currently only applied after the transition. That is a pity because child participation could lead to more engagement and motivation for learning by children and to feedback for teachers to improve their pedagogical practice [15].

In the literature there is consensus that communication between primary and secondary schoolteachers is vital to improve the transition and can be improved via direct contact between teachers [32,51,58]. This was confirmed by the mentors in our study. Despite the problems and shortcomings, the mentors in the focus groups said that they were satisfied with the quality, reliability and usability of the information they received through directly personal contact, for example via ambassadors. Personal contact appeared to be the most complete and the most useful for allocating the student to the correct level. However, mentors were less satisfied about aspects of the preparation for secondary school, the quality of the school-advice, the comprehensiveness of the educational-report made by the primary school, and a lack of time that teachers of primary school have to meet the mentor for a conversation or for feedback.

Because the transition does not start when the children start at secondary school, primary schoolteachers need to know how it works at secondary school, and vice versa, to prepare the children adequately [25]. According to the mentors, primary schoolteachers do not have a clear idea of secondary education witch then impacts on the preparation for secondary education at primary school. As an example: the children are expected to be prepared to work independently in secondary education [25]. Nevertheless, the mentors noted that, despite the fact that the children are becoming more assertive, unfortunately they are also more and more dependent on the help of the mentor to solve their social problems. The mentors at secondary school have to navigate their students more and more to navigate their social skills. They blame this partly on the increasing use of social media, which tends to limit direct, personal communication between the children, and partly on a failure by some parents to raise their children with proper social skills and behavior.

The content of the educational-report is incomplete at the moment the classes are formed, because the privacy law does not allow the files to be transferred before 1 August, the official start of the new school year. This may lead to strategic behavior with abuse of the administrative process, as information which may have a negative impact on the placement is sometimes deliberately withheld from the secondary school. This is undoubtedly not the intention of the privacy law.

Not every school-advice is as reliable as it should be. Some parents try to influence the school-advice by withholding information or trying to force to primary school to give an advice for a higher level because they think their child deserves a chance at that level. Some primary schools systematically give an (too) high advice because they cannot properly estimate what is asked of the children at secondary school, or because they think it is sad for a child to enter at the lowest level of secondary education. Instead of a successful transition desired this may lead to frustration for the children, poor performances and, ultimately, to dropping down to a lower level.

Finally, it seems that teachers from grade six and mentors from grade seven lack the time to connect and meet with each other for a conversation before the transition or to provide feedback after the transition. They receive insufficiently support to create and maintain the contacts that they acknowledge as being the most valuable.

The school mentors we interviewed, indicated how the transition from primary to secondary school could be improved. They mentioned aspects such as a ‘warm transfer’ where the teacher of the final year in primary education meets with the mentor of the secondary school to discuss the student, preparation for the transition, knowledge of each other's educational practice, the school advice and involving children and parents actively in this advice. To assess the extent to which these conclusions are broadly supported – that is, to assess the inter-subjectivity, credibility and transferability of our findings – we compared the conclusions of our study to the findings about the same topics by the Dutch Inspectorate of Education [27,28].

The national study by the Inspectorate indicate that our results are not limited to the southeast of the Netherlands. The Inspectorate claims in their annual report on the condition of education in 2019 [28]. That better cooperation between primary and secondary education would ensure that students end up in the right place. According to the Inspectorate, primary schools and primary school teachers often have their own ideas about the options for guidance and support for secondary schools, which they include in their advice. Sometimes children are only "informed" about the advice after the fact. The Inspectorate advises schools to learn how to handle pressure from parents to give a higher school advice or to place a child in a higher type of secondary education. The results of this paper confirm the perceptions of the Inspectorate.

### 5.1 Limitations

This study had some limitations that need to be acknowledged. Our study was limited in size and, by its focus on the mentor’s perspective, limited in the diversity of stakeholders involved in the transition process. The study was restricted to the experiences of mentors in the Netherlands who have a non-committed role and remain free to decide for themselves whether and how they use the information they receive about the children in their class. This can lead to differences in the use of information and the approach taken and may limit the ability to generalize the findings to other populations. The study was also limited by the regional location of the schools. A larger scale study is recommended to further explore the validity of our findings.

## 6 Recommendations

This study is innovative because – to the best of our knowledge – it is one of the first to present the opinions about the transition from primary to secondary education of mentors in secondary education who work directly with the children. The recommendations were derived from their experiences and expectations. The comments of the interviewed mentors, were usually not about grades or test results but concern the communication between primary and secondary school, the children’s lack of skills to be prepared for secondary education, the role of the parents and the absence of the children involved in the preliminary stage of the transition process. These comments resulted in the recommendations below.

Mentors often function autonomously, but are also part of the school organization where they work. Therefore our recommendations are aimed at both the individual mentors and the school organization. We successively describe the recommendations to the mentor, the recommendations to the schools and then the need for further study.

• Recommendations to mentors

Because the transition process is ultimately about the ongoing positive academic and social emotional development of the child, a mentor should be able understand and respond to the issues children are concerned about by the children themselves. We argue that all children should have a personal conversation with their new mentor before they make the transition to secondary education.

The problems between primary and secondary school with the school-advice can only be tackled when all stakeholders are working together well. To prevent unpleasant surprises for children, both primary and secondary schools need to agree and to be transparent about what skills are required in secondary education, at what level, and about what skills are missing. We recommend that the teachers not only communicate with other teachers but also with the parents and the children in a more intensive collaboration to provide all stakeholders with feedback about their experiences after the transition. To make this possible, teachers on both sides of the transition need to be given adequately time.

• Recommendations to the schools

Parents' support before, during and after the transition, is a crucial tool for children to get used to the changing learning environment and learn to adapt to the pedagogical approach at secondary school [32,38]. In accordance with the advice of the Education Council, we advise schools to create opportunities to involve parents as partners in education.

Family support can improve the transition: children stand to benefit when schools and parents see eye-to-eye [1], and both teachers and parents have the opportunity to support children to develop their social and emotional skills [9]. We agree with Elias et al. [9]. They recommend that schools use suitable programs to train in these skills, and advises parents to help their children, to deal with questions and to develop conversation and listening skills, along with courtesy and empathy. Unfortunately, the results of the current study show that these recommendations have yet to be implemented. We would like to advice schools not only to provide, but also to evaluate the impact of the support of all stakeholders involved in the process, including the children.

• Further study

Further study about good practices, such as the initiative where personal information is collected at the feeding school of each future student (combined with feedback from the receiving school via personal contact) is recommended. This initiative seems to have the possibility of preventing placement problems resulting in fewer re-allocation of students in different tracks of secondary education.

## Author contribution statement

Marlau van Rens: Conceived and designed the experiments; Performed the experiments; Analyzed and interpreted the data; Contributed reagents, materials, analysis tools or data; Wrote the paper.

Wim Groot: Conceived and designed the experiments; Analyzed and interpreted the data; Wrote the paper.

## Data availability statement

Data will be made available on request.

## Declaration of competing interest

The authors declare that they have no known competing financial interests or personal relationships that could have appeared to influence the work reported in this paper.

Supplementary content related to this article has been published online at [URL].

## References

[1] Anderson L., Jacobs J., Schramm S., Splittgerber F. (2000). School transitions: beginning of the end or a new beginning?. Int. J. Educ. Res..

[2] Berki B., Málovics G., Remus C. (2021).

[3] Boone S., Demanet J. (2020). Track choice, school engagement and feelings of perceived control at the transition from primary to secondary school. Br. Educ. Res. J..

[4] Bokhorst C., Sumter S., Westenberg M. (2010). Social support from parents, friends, classmates, and teachers in children and adolescents aged 9 to 18 years: who is perceived as most supportive?. Soc. Dev..

[5] Cretan R., Turnock D. (2008). Romania’s Roma population: from marginality to social Integration. Scottisch Geographical J..

[6] Coffey A. (2013). Relationships: the key to successful transition from primary to secondary school?. Improv. Sch..

[7] De Witte K., Rogge N., Dropout from secondary education: all’s well that begins well, TIER Working Paper Series, TIER WP 14/11 (2014) 978 94 003 0077 4. DOI: 10.111/ejed.12001. (working paper).

[8] Dinklo I. (2006). Fabels en feiten over kwalitatieve onderzoeksresultaten. (Myths and facts about qualitative research results. Kwalon.

[9] Elias M., Patrikakou E., Weissberg R. (2007). A competence- based framework for parent- school- community partnerships in secondary schools. Sch. Psychol. Int..

[10] Ellerbrock C., Abbas B., DiCicco M. (2014). Developmentally responsive teacher practices across the middle-to-high-school transition. J. Res. Edu..

[11] European Commission/EACEA/Eurydice (2018). https://op.europa.eu/en/publication-detail/-/publication/9f68b65e-cc36-11e8-9424-01aa75ed71a1.

[12] Evangelou M., Taggart B., Sylva K., Melhuish E., Sammons P., Siraj- Blatchford I. (2008). https://www.semanticscholar.org/paper/What-makes-a-successful-transition-from-primary-to-Evangelou-Taggart/bf4cf879ddfccc986cf4c18d49b322ef64b38046.

[13] Evers J. (2023).

[14] Faas D., Haijsoteriou C., Angelides P. (2014). Intercultural education in Europe: policies, practices and trends. Br. Educ. Res. J..

[15] Ferguson D., Handreddy A., Draxton S. (2011). Giving pupils a voice as a strategy for improving teacher practice. Lond. Rev. Educ..

[16] Fink A. (2008).

[17] Frederiksen K., Rhodes J. (2004). The role of teacher relationships in the lives of students. N. Dir. Youth Dev..

[18] Ganeson K., Ehrich L. (2009). Transition into high school: a phenomenological study. Educ. Philos. Theor..

[19] Glaser B., Strauss A. (1967). http://www.sxf.uevora.pt/wp-content/uploads/2013/03/Glaser_1967.pdf.

[20] Glaser B.G. (1978).

[21] Glaser B.G. (1998).

[22] Government of the Netherlands Appropriate education at primary school. Available at: https://www.government.nl/topics/primary-education/appropriate-education-at-primary-school (Accessed: 26 March 2023).

[23] Guba E., Lincoln Y. (1994). Handbook of Qualitative Research.

[24] Hanewald R. (2013). Transition between primary and secondary school: why it is important and how it can be supported. Australian J. Teacher Edu..

[25] Hopwood B., Hay I., Dyment J. (2016). The transition from primary to secondary school: teachers’ perspectives. Aust. Educ. Res..

[26] Humphrey N., Ainscow M. (2006). Transition club: facilitating learning, participation and psychological adjustment during the transition to secondary school. Eur. J. Psychol. Educ..

[27] Inspectie van het onderwijs (2019). https://www.onderwijsinspectie.nl/documenten/rapporten/2019/04/10/rapport-de-staat-van-het-onderwijs-2019.

[28] Inspectie van het onderwijs (2018). https://www.onderwijsinspectie.nl/documenten/rapporten/2018/04/11/rapport-de-staat-van-het-onderwijs.

[29] Jallade J.P. (1992). Undergraduate higher education in Europe: towards to a comparative perspective. Eur. J. Educ..

[30] Jindal-Snape D., Symonds J.E., Hannah E.F.S., Barlow W. (2021). Conceptualising primary-secondary school transitions: a systematic mapping review of worldviews, theories and frameworks. Fronti. Edu..

[31] Kelle U. (2006). Combining qualitative and quantitative methods in research practice: purposes and advantages. Qual. Res. Psychol..

[32] Korpershoek H., Beijer C., Spithoff M., Naaijer H., Timmermans A., van Rooijen M., Vugteveen J., Opdenakker M. (2016). https://www.nro.nl/sites/nro/files/migrate/Eindrapport-405-14-402-project-1-Reviewstudie-naar-de-po-vo-en-de-vmbo-mbo-overgang.pdf.

[33] Lavrijsen J., Nicaise I., Wouters T. (2013). https://kuleuven.limo.libis.be/discovery/fulldisplay?docid=lirias1897495&context=SearchWebhook&vid=32KUL_KUL:Lirias&lang=en&search_scope=lirias_profile&adaptor=SearchWebhook&tab=LIRIAS&query=any,contains,LIRIAS1897495&offset=0.

[34] Mason J., Danby S. (2011). Children as experts in their lives: child inclusive research. Child Indicators Res..

[35] McGee C., Ward R., Gibbons J., Harlow A. (2004). https://scholar.google.com/scholar?hl=en&as_sdt=0%2C5&q=McGee%2C+C.%2C+Ward%2C+R.%2C+Gibbons%2C+J.+&+Harlow%2C+A.+%282004%29.+Transition+to+secondary+school%3A+a+literature+review.+&btnG=.

[36] Naaijer H.M., Spithoff M., Osinga M., Klitzing N., Korpershoek H., Opdenakker M.-C. (2016). De overgang van primair naar voortgezet onderwijs in internationaal perspectief: een systematische overzichtsstudie van onderwijstransities in relatie tot kenmerken van verschillende Europese onderwijsstelsels. (The transition from primary to secondary education in an international perspective: a systematic overview study of educational transitions in relation to characteristics of different European education systems. GION onderwijs/onderzoek.

[37] Noddings N. (2005). Identifying and responding to needs in education. Camb. J. Educ..

[38] Onderwijsraad (2010). https://www.onderwijsraad.nl/publicaties/adviezen/2010/02/17/ouders-als-partners.

[39] Peel K.L. (2021). Professional dialogue in researcher-teacher collaborations: exploring practices for effective student learning. J. Educ. Teach..

[40] Poorthuis A. (2012). https://dspace.library.uu.nl/handle/1874/257812.

[41] Po-Raad, Vo-Raad en A.V.S. (2011). https://www.vanponaarvo.nl/wp-content/uploads/2014/10/onderzoeksrapport-overstap-po-vo-oberon.pdf.

[42] Powell R., Smith R., Jones G., Reakes A. (2006). https://www.nfer.ac.uk/media/2243/wtn01.pdf.

[43] Rice F., Frederickson N., Seymour J. (2011). Assessing pupil concerns about transition to secondary school. Br. J. Educ. Psychol..

[44] Rodrigues R.G., Meeuwisse M., Notten T., Severiens S.E. (2018). Preparing to transition to secondary education: perceptions of Dutch pupils with migrant backgrounds. Educ. Res..

[45] Rupsiene L., Kucinskiene R. (2005). https://files.eric.ed.gov/fulltext/ED490657.pdf.

[46] Smeets E., van Kuijk J., Driessen G. (2014).

[47] Strauss A. (1987).

[48] Tilanus C. (1997).

[49] Tobbell J. (2003). Students’ experiences of the transition from primary to secondary school. Educ. Child Psychol..

[50] Tobbell J., O’Donnell V. (2013). The formation of interpersonal learning relationships in the transition from primary to secondary school: students, teachers and school context. Int. J. Educ. Res..

[51] Topping K. (2011). Primary- secondary transition: differences between teachers’ and children’s perceptions. Improv. Sch..

[52] Van Rens M., Haelermans C., Groot W., Maassen van den Brink H. (2018). Facilitating a successful transition to secondary school: (how) does it work? A systematic literature review. Adoles. Res. Rev..

[53] Van Rens M., Haelermans C., Groot W., Maassen van den Brink H. (2019). Girls’and boys’perceptions of the transition from primary to secondary school. Child Indic. Res..

[54] Van Rens M., Groot W., Haelermans C. (2020). (How) does information provided by children affect the transition from primary to secondary school?. Child Indic. Res..

[55] Van Rens M. (2020).

[56] Van Rooijen M., Korpershoek H., Vugteveen J., Timmermans A., Opdenakker M. (2016). https://research.rug.nl/en/publications/overgangen-en-aansluitingen-in-het-onderwijs-deelrapportage-2-emp.

[57] Van Ryzin M. (2010). Secondary school advisors as mentors and secondary attachment figures. J. Community Psychol..

[58] West P., Sweeting H., Young R. (2010). Transition matters: pupils’ experiences of the primary- secondary school transition in the West of Scotland and consequences for well-being and attainment (2010). Res. Pap. Educ..

[59] Zeedijk M., Gallacher J., Henderson M., Hope G., Husband B., Lindsay K. (2003). Negotiating the transition from primary to secondary school. Perceptions of pupils, parents and teachers. Sch. Psychol. Int..

